# Quantification of Virus Particles Using Nanopore-Based Resistive-Pulse Sensing Techniques

**DOI:** 10.3389/fmicb.2016.01500

**Published:** 2016-09-22

**Authors:** Lu Yang, Takatoki Yamamoto

**Affiliations:** Department of Mechanical Engineering, School of Engineering, Tokyo Institute of TechnologyTokyo, Japan

**Keywords:** virus particle, quantification, resistive-pulse sensing, out-of-plane nanopore, in-plane nanopore

## Abstract

Viruses have drawn much attention in recent years due to increased recognition of their important roles in virology, immunology, clinical diagnosis, and therapy. Because the biological and physical properties of viruses significantly impact their applications, quantitative detection of individual virus particles has become a critical issue. However, due to various inherent limitations of conventional enumeration techniques such as infectious titer assays, immunological assays, and electron microscopic observation, this issue remains challenging. Thanks to significant advances in nanotechnology, nanostructure-based electrical sensors have emerged as promising platforms for real-time, sensitive detection of numerous bioanalytes. In this paper, we review recent progress in nanopore-based electrical sensing, with particular emphasis on the application of this technique to the quantification of virus particles. Our aim is to provide insights into this novel nanosensor technology, and highlight its ability to enhance current understanding of a variety of viruses.

## Introduction

Viruses are a major cause of infectious diseases. As such, they hold great significance in virological and immunological research and have extensive applications in clinical diagnosis and therapy. Determining both the dimensions and number of viruses is extremely important in many applications, such as the production of virus-based vaccines and therapeutic agents; hence, quantitative detection of viruses is becoming increasingly important. From a microbiological perspective, viruses are infectious agents that replicate only inside host cells. Single virus particles, also known as virions, generally consist of either double-stranded or single-stranded genetic molecules (DNA or RNA) surrounded by a protein shell called a capsid. In some cases, the capsid is enclosed within an outer lipid envelope. Several techniques are available for virus quantification (Heider and Metzner, 2014), including (1) determination of infectivity levels via plaque-forming and 50% tissue culture infectious-dose assays; (2) detection of virus proteins via antibody-antigen binding (e.g., enzyme-linked immunosorbent assays); (3) quantification of the viral genome using polymerase chain reaction (PCR), quantitative reverse-transcription (qRT)-PCR, and a range of metagenomic techniques; and (4) simultaneous determination of the presence of both stained proteins and nucleic acids using flow cytometry. Although these methods offer high specificity, drawbacks such as time-consuming and complicated procedures still limit their widespread utilization; thus, the development of new sensing technologies is highly desired.

The typical physical dimensions of individual virus particles range from several tens to hundreds of nanometers. Viruses are thus a type of functional nanoparticle. Current widely used nanoparticle-sensing technologies should therefore be suitable for the characterization of virus particles, since to some extent, virus particles can be treated as soft nanoparticles. Theoretically, these technologies should detect all virus and virus-like particles, regardless of their infectivity, providing important complementary information (e.g., the ratio of total to infective virus particles). Nanoparticle-sensing technologies can be broadly classified into two categories: visualization-based and non-visualization-based techniques. Visualization-based techniques include transmission electron microscope (TEM) (Schatten, 2011; Harris, 2014) and atomic force microscope (Kasas and Thomson, 1997; Ohnesorge et al., 1997; Allison et al., 2010; Mateu, 2012), in which the size, shape, and concentrations of viruses are determined visually. However, these techniques are somewhat low-throughput, labor-intensive, and require high-level technical expertise to operate the costly associated equipment. Additionally, special treatment of samples is required, which sometimes results in inaccurate measurements due to aggregation and deformation of virus particles. Non-visualization-based techniques are based on light scattering analysis, including dynamic light scattering (DLS) (Driskell et al., 2011) and nanoparticle tracking analysis (NTA) (Kramberger et al., 2012; Nikolai et al., 2015). DLS measures the hydrodynamic Stokes-Einstein radius of particles undergoing Brownian motion by light scattering generated by an incident laser light source. The main problems associated with this technique are low sensitivity and resolution caused by detection of the ensemble average of particles and unsuitability for polydispersed samples. In contrast to DLS, NTA is suitable for identifying and tracking individual particles. However, the refractive index of the sample must be distinctive from that of the surrounding medium, and NTA often overestimates the size of particles compared with TEM.

Development of a rapid, high-throughput, real-time, label-free, sensitive, accurate, and (hopefully) miniaturized system to detect single virus particles must address the problems and limitations associated with the aforementioned technologies. One promising approach is the use of electrical detection techniques based on probing changes in resistance and/or capacitance using a nanoscale constriction. Over the last two decades, these techniques have demonstrated great capabilities of sensing a wide range of biomolecules (Yurt et al., 2012; Harms et al., 2015a), driven by significant advances in nanofabrication and electronics technologies. Electrical detectors utilizing a variety of sensing principles are available; nevertheless, we focus here primarily on the popular resistive-pulse sensing (RPS) detector, which is based on resistance measurement. We review recent progress and discuss future perspectives for this emerging electrical sensing technique, with the goal of providing insights into the key issues of reliable and effective quantification of individual viruses.

## Basic theory of nanopore-based RPS

**Figure 1:**
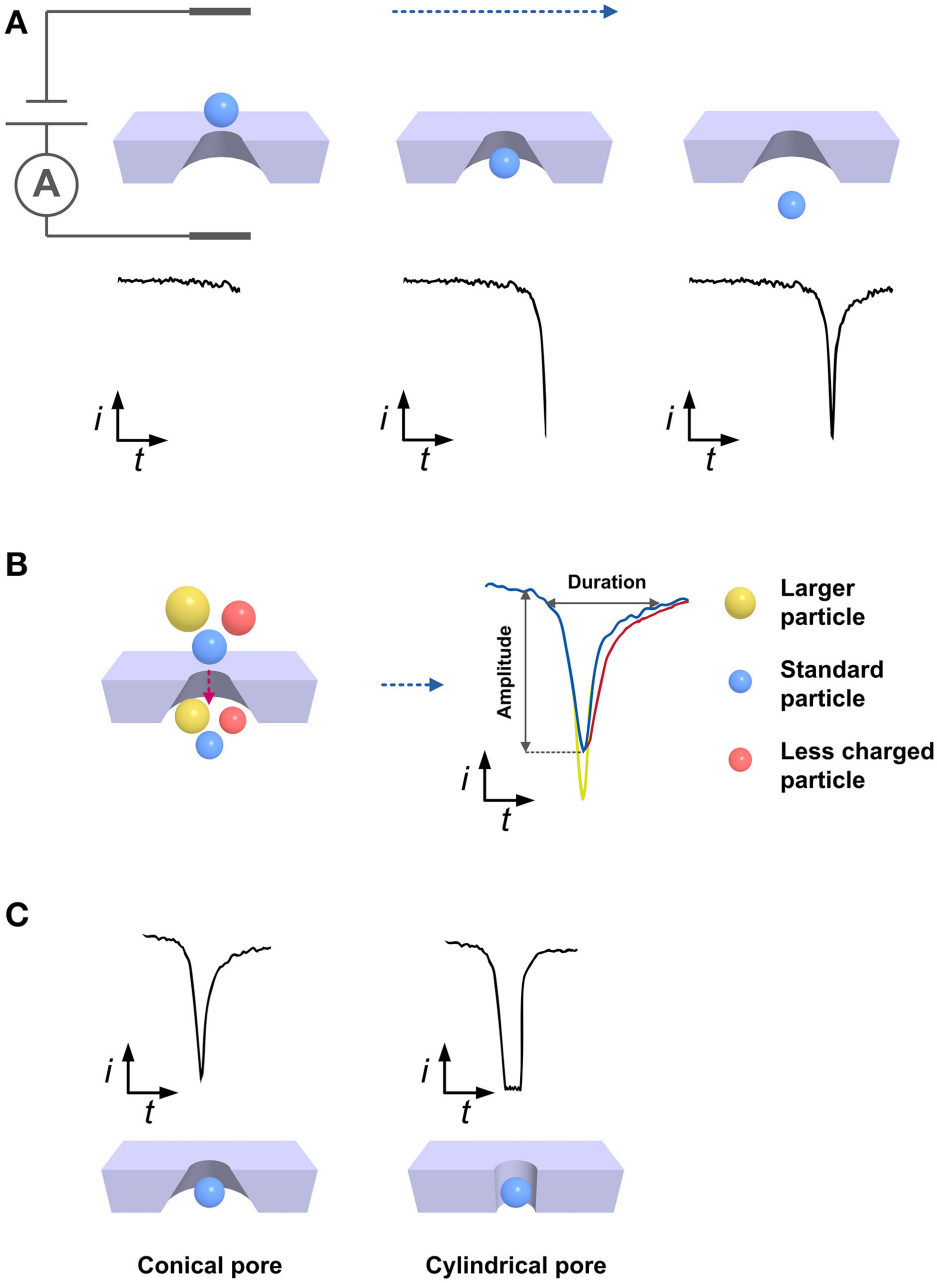
**Schematic illustrating basic principles of nanopore-based RPS technique. (A)** Current changes during particle translocation across the nanopore. **(B)** Differences in pulses resulting from the translocation of particles with different sizes or surface charges. The yellow sphere is larger than the blue one and they carry the same amount of surface charge. The red sphere is less charged compared to the blue one and they have the same size. **(C)** Effect of pore's geometry on pulse shape. Conical and cylindrical pores commonly give rise to the asymmetric and symmetric pulses.

The origin of RPS dates back to Coulter counting technique, patented in 1953. The basic apparatus comprises two separate electrolyte solution-containing chambers connected by a small pore with dimensions comparable to the analyte of interest. As shown in Figure 1A, an electrical current is generated when a constant electric potential is applied between two electrodes placed on each side of the pore. As electrolyte buffer carrying insulated or poorly conductive particles passes through the chambers, translocation of particles across the pore causes a transient increase in pore resistance and a corresponding drop in current, recorded as a series of pulses. In the simplest case, where a spherical particle passes through a cylindrical pore, the relative change in resistance is described by:

$$\begin{array}{l} \frac{△R}{R}=\frac{d^{3}}{D^{2}L} \end{array}$$

where *d* and *D* are the particle and pore diameters, respectively. *L* is the pore length. The particle translocation process is driven predominantly by convection flow and electrokinetic flow, including electrophoretic and electroosmotic flow. According to the Nernst-Planck equation, the translocating particle flux, *J*, referring to the number of particles passing through a unit area of the pore per unit time, is expressed as:

$$\begin{array}{l} J\;\approx \;J_{eph}\;+\;J_{eo}\;+\;J_{pd}, \end{array}$$

where *J*_*eph*_, *J*_*eo*_, and *J*_*pd*_ represent the electrophoretic, electroosmotic, and pressure-driven fluxes, respectively (Willmott and Smith, 2014). The sum of electrokinetic fluxes is given by:

$$\begin{array}{l} J_{eph}\;+\;J_{eo}\;\approx \;\frac{C\varepsilon }{\eta }\left(\zeta_{particle}\;-\;\zeta_{pore}\right)E, \end{array}$$

where *C* represents the particle concentration, ε and η represent the permittivity and viscosity of the electrolyte, respectively, ζ represents the Zeta potential of the subscripted surface, and *E* represents the electric field strength. Figure 1B shows that the amplitude of the pulse is directly proportional to the particle volume. The pulse duration and frequency can also be used to infer information regarding the particle concentration and Zeta potential, which is related to the surface charge of the particle and serves as an indicator of colloidal system stability (Kozak et al., 2011).

This particle-by-particle readout technique provides a wealth of information while requiring a lower sample concentration (~10^7^ particles/mL), smaller sample volume (~40 μL), and less operating time (~10 min) than traditional sensing techniques. Consequently, PRS has been employed for detection of DNA, proteins, viruses, bacteria, particles for drug delivery system (e.g., emulsions and liposomes), extracellular vesicles, and inorganic and polymeric nanoparticles (Venkatesan and Bashir, 2001; Howorka and Siwy, 2009; Oukhaled et al., 2012; Platt et al., 2012; Colby et al., 2013; Somerville et al., 2013; Stoloff and Wanunu, 2013; Willmott et al., 2013). The application of RPS for virus particle analysis is introduced in the following section.

## Applications of RPS for virus detection

PRS detectors are generally composed of sensing electronics and nanopores that enable every single nanoparticle in a sample to pass through them one by one. The sensing electronics commonly include Ag/AgCl electrodes, a current amplifier, filter, and data acquisition unit, whereas the nanopores can vary distinctly in dimension, material, geometry, and structure. Although some biological pores made of natural proteins embedded in lipid bilayer membranes exist, the lack of robustness and extremely small pores (<5 nm) make them unsuitable for virus detection (Haque et al., 2013; Ying et al., 2014). Hence, we focus mainly on synthetic nanopores. As shown in Figure 1C, some pores are cylindrical and generate a symmetric pulse, although the overwhelming majority of pores are conical in shape and produce an asymmetric pulse that indicates the translocation direction (Davenport et al., 2013). Nanopores generally fall into one of two prototype categories according to the pore orientation relative to the substrate: out-of-plane or in-plane. Out-of-plane nanopores are perpendicular to the substrate surface and independent of the fluid chamber, which is commonly fabricated on a thin membrane supported by an insulating substrate. In contrast, in-plane nanopores are parallel to the substrate surface, which is embedded into a micro/nanofluidic channel as a built-in unit.

### Out-of-plane nanopore sensors

#### Solid-state nanopores

Nanopores were utilized to analyze virus particles as early as 1977 (DeBlois and Wesley, 1977). Using submicron-diameter polycarbonate pores, DeBlois and Wesley measured the size of several type C oncornaviruses (Rauscher murine leukemia [122.3 ± 2 nm], simian sarcoma [109.7 ± 3 nm], Mason-Pfizer monkey [140.0 ± 2.5 nm], RD-114 [115 ± 5 nm], and feline leukemia [127.4 ± 2 nm]) and T2 bacteriophage (5.10 ± 0.15 × 10^−16^ cm^3^) by comparing their pulse height to that of standard polystyrene latex beads. In addition, viruses were counted not only by comparing viruses and latex beads, but also by measuring the flow rates, which is related to the concentration. They reported achieving a lower practical count limit of 5 × 10^7^ particles/mL. The same group subsequently compared measurements of other viruses (including Tipula iridescent virus, nuclear polyhedrosis viruses of the gypsy moth and European pine sawfly, Sindbis virus, and vesicular stomatitis virus) using light-scattering and electron microscopy to RPS measurements (DeBlois et al., 1978; Feuer et al., 1978). The results obtained using the different techniques were in general agreement.

**Figure 2:**
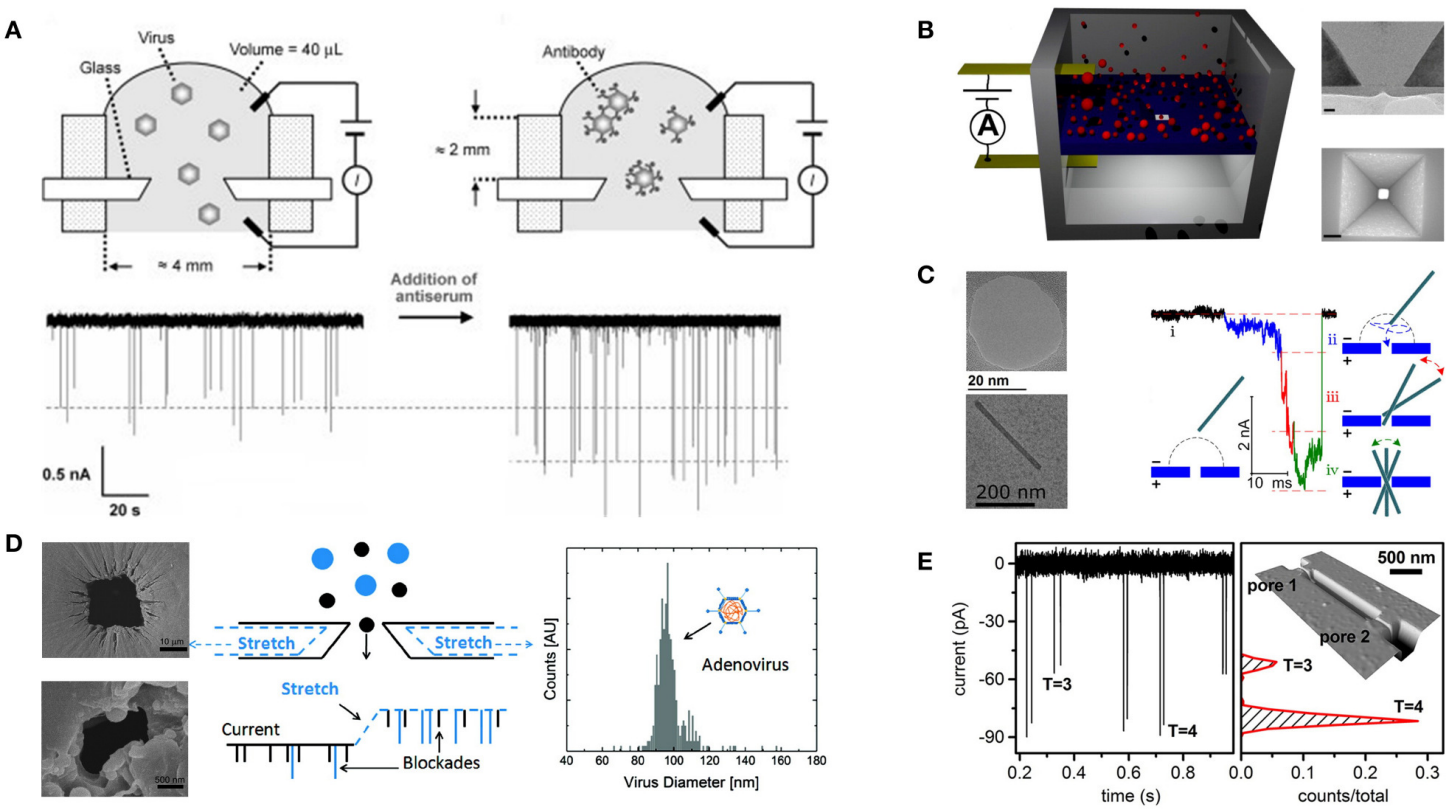
**Examples of RPS being applied to virus detection. (A)** Characterization of the binding of antibodies to virus particles by detecting virions passing through a sub-micrometer glass pore. The binding of antibodies to the virus leads to an increase in the pulse amplitude. Reprinted with permission from Uram et al. (2006). Copyright Wiley-VCH Verlag GmbH & Co. KGaA, Weinheim. **(B)** Pyramidal-shaped solid-state nanopores. Images on the right side are TEM cross section image of a 40 nm nanopore and SEM top-view image of a 120 × 120 nm nanopore. Adapted with permission from Arjmandi et al. (2014). Copyright 2014 American Chemical Society. **(C)** Translocation dynamics of the rod-shaped virus. TEM images on the left side show a typical 30 nm solid-state nanopore and a single TMV particle. Adapted with permission from Wu et al. (2016). Copyright 2016 American Chemical Society. **(D)** Particle size distribution of adenovirus detected by TRPS. SEM images represent the large and small pore openings of a thermoplastic polyurethane membrane. Adapted with permission from Vogel et al. (2011). Copyright 2011 American Chemical Society. **(E)** Detection of HBV capsids (*T* = 3 and *T* = 4 refer to capsids with outer diameter of 32 and 35 nm, respectively) passing through in-series nanopores. Reprinted with permission from Harms et al. (2015b). Copyright 2014 American Chemical Society.

Recently, Uram and coworkers investigated the interactions between *Paramecium bursaria* chlorella virus (diameter ~190 nm) and specific antibody using RPS as shown in Figure 2A (Uram et al., 2006). A conical pore with a 650-nm diameter was fabricated on a glass cover slide using a femtosecond-pulsed laser. They estimated the maximum number of antibodies binding to *Paramecium bursaria* chlorella virus and attempted to elucidate the kinetics of the antibody-virus interaction by simply detecting changes in the pulse amplitude after adding antibodies to the virus samples. Zhou and coworkers (Zhou et al., 2011) succeeded in discriminating between hepatitis B virus (HBV) capsids assembled from different numbers of dimers. They fabricated a 40-nm track-etched conical pore in a poly(ethylene terephthalate) membrane. Notably, the surface of this nanopore was covalently modified with triethylene glycol to minimize capsid adsorption and suppress electroosmotic flow within the pore. Arjmandi and coworkers constructed pyramidal-shaped pores as shown in Figure 2B to detect human immunodeficiency virus and Epstein-Barr virus (Arjmandi et al., 2012, 2014). They fabricated pores of 20–500 nm in size on a silicon membrane using electron beam lithography followed by anisotropic wet etching using potassium hydroxide. Their major contribution was that they established a Zeta potential measurement method based on the translocation velocity of particles. Their work revealed that the results of Zeta potential measurements agreed well with DLS measurements.

In addition to a great number of studies focusing on sphere viruses, due to their well-defined shapes, the translocation of rod-shaped virus particles across a nanopore has also been investigated. McMullen and coworkers examined the basic physics of translocation of the stiff filamentous virus fd (6.6 × 880 nm) (McMullen et al., 2014). TEM-drilled nanopores of 12–50 nm in diameter were formed in silicon nitride membranes. Using these pores, the authors could distinguish translocation of viruses through the nanopore from side-on collisions of the viruses with the sidewall of the nanopore by comparing the amplitude and duration of the corresponding pulses. Wu and colleagues (Wu et al., 2016) used similar nanopores to observe and simulate the translocation of tobacco mosaic virus (18 × 300 nm). They found that tobacco mosaic virus has to rotate to pass through the nanopore after interacting with the pore surface. Figure 2C shows that this rotation significantly influences the current signal.

Overall, solid-state nanopores with fixed pore sizes ranging from several nanometers to sub-micrometer have been fabricated in a broad range of materials (e.g., glass, silicon, silicon nitride, polymers) using a variety of state-of-the-art nanofabrication techniques (e.g., electron beam and focused ion beam milling) (Miles et al., 2013). A tremendous number of advantages are recognized, such as the ease of manufacturing and introducing surface modifications, nanopore size comparable to virus size range, exceptional robustness, and increased signal-to-noise ratios. Currently, these nanopore sensors play a dominant role in virus sensing, suggesting that their use will only increase in the future.

#### Tunable elastomeric nanopores

The aforementioned nanopores with fixed pore sizes are not suitable for polydispersed sample measurements, resulting in a slightly limited detectable size range. To overcome this limitation, size-tunable nanopores that enable *in situ* adjustment of nanopore size to match that of the analyte were introduced in 2011, leading to improved measurement sensitivity (Blundell et al., 2015; Weatherall, 2015). This technology, which is designated tunable resistive-pulse sensing (TRPS), was developed almost exclusively by Izon Science Ltd. (Christchurch, New Zealand). The nanopores are fabricated on thermoplastic polyurethane membranes using tungsten needles. The pore size can be finely tuned by stretching and relaxing the membrane in a biaxial and reversible manner. These physically and chemically stable nanopores with tunable pore sizes both extend the analysis range and make it possible to recover clogged pores by simply stretching them. Izon Science has released several commercially available products (“qNano” and “qViro-X”) containing compactly integrated nanopores with actuation and electronic components. In addition, qNano is equipped with a variable pressure module that can generate external positive or negative pressure to facilitate or hinder particle passage to optimize the translocation rate. The qNano system is also equipped with data collection and analysis software. Measurement protocols have been established to simultaneously elucidate the particle size, concentration, and Zeta potential (Kozak et al., 2012; Vogel et al., 2012; Eldridge et al., 2014). The results of TPRS studies have been comparable to measurements using other techniques, such as TEM, DLS, and NTA (Anderson et al., 2013).

In analyses of both synthetic and biological particles, TRPS has demonstrated substantial benefits, including portability, simplicity, and versatility (Roberts et al., 2012; Adela Booth et al., 2013; Yu et al., 2014; Anderson et al., 2015; Lane et al., 2015). With respect to virus analysis, Vogel and colleagues demonstrated the feasibility of sizing adenovirus (70–90 nm) using qNano as shown in Figure 2D (Vogel et al., 2011). They determined the size distribution of purified adenoviruses and calculated their modal diameter (96.5 ± 15 nm). Farkas and coworkers counted rotavirus (75 nm) using both qRT-PCR and qNano to evaluate the purification of the viruses by size-exclusion chromatography (Farkas et al., 2013). Akpinar and Yin counted vesicular stomatitis virus (70 × 200 nm) using both TRPS and a plaque assay (Akpinar and Yin, 2015). The average total to infectious particle ratio was calculated as 2.91 ± 1.42. In addition, the mean equivalent particle diameter of this bullet-shaped virus, which reflects the diameter of a sphere with an equal volume, was measured as 107.8 and 111.8 nm by TRPS and TEM, respectively.

Despite these applications, to date only a few studies have reported using TRPS for quantification of virus particles, perhaps due to insufficient familiarity with TRPS, which was invented only a few years ago and remains in an early stage of development. Another reason is that the lowest detection limit of TRPS is reportedly only 70 nm when the smallest nanopore (NP100; 100 nm pore diameter) is used. This is insufficient for detecting the majority of viruses with a size in the 10 s of nm or viruses with strongly anisotropic dimensions (e.g., bacteriophages and rod-shaped viruses). There is thus a demand for further decreases in pore size to improve detection limits.

### In-plane nanopore sensors

In-plane naopores are compactly integrated into micro/nanofluidic devices, leading to enhanced portability and fluid control, lower sample consumption, ease of observing particle translocation optically, and improved mass transfer of analytes to the nanopore. Moreover, incorporating multiple pores in series or in parallel can increase the throughput and the device functionality (Fraikin et al., 2011; Haywood et al., 2015). However, the number of relevant studies is low. A systematic method for quantitative measurements of particle size, concentration, as well as surface charge using in-plane nanopores is needed.

Harms and coworkers fabricated a nanochannel with two nanopores in series to detect HBV capsids as shown in Figure 2E (Harms et al., 2011). The nanochannel and nanopores were made on a silicon wafer using electron beam lithography and a two-step thermal oxidation process. The nanochannel and nanopore dimensions were 1000 × 50 × 1000 nm and 50 × 50 × 40 nm (width × depth × length), respectively. A pulse pair representing a single capsid passing through two pores successively exhibited almost identical amplitudes. The migration time needed for a capsid traveling from the first pore to the second pore was calculated from pairs of adjacent pulses, which were used to estimate the electrophoretic mobility of HBV capsids. The authors also used focused ion beams to make nanochannels and nanopores on a glass substrate to determine the electrophoretic mobility of HBV capsids with different molecular weights and to monitor the assembly process (Harms et al., 2015b,c).

## Conclusions and outlook

In conclusion, nanopore-based electrical sensing techniques have experienced significant growth as emerging yet promising platforms for nanoparticle detection, driven by dramatic advances in nanotechnology. Nanopore-based electrical sensing provides excellent capabilities of quantifying virus particles in a real-time, label-free, high-throughput, and particle-by-particle manner.

However, current trends suggest that some challenges still remain and need to be overcome if the range of practical applications is to widen. First, improvements in pore fabrication and signal readout are needed. Reproducible fabrication and improved readout capabilities will enhance measurement reproducibility, increase sensitivity, and lower the detection limits. Introducing surface modifications or coatings within nanopores is necessary as well, as this can minimize non-specific adsorption and pore clogging. Second, even though research indicates that the accuracy of electrical sensing is comparable to that of DLS and TEM technologies, the combination with other non-electrical sensing techniques during measurements will be an interesting trend. For example, it was reported that simultaneous electrical and optical analysis can provide “double-checked” results (Liu et al., 2014; Hauer et al., 2015). Finally, a greater diversity of viruses should be analyzed in terms of viron size, concentration, and surface charge. Determination of the total to infectious particle ratio and the kinetics of virus-antibody binding would be of particular interest. For diagnostic applications, strict steps should be taken to avoid false-positive and false-negative results. If advances enable nanopore-based methods to provide better performance than conventional biochemical assays, they could be adapted for routine clinical use.

The most obvious advantage of nanopore-based electrical methods is the possibility of detecting unknown and new species of viruses. Unknown viruses are intrinsically difficult to detect using traditional methods because information regarding the genome or membrane proteins is necessary prior to the design of PCR primers or antibodies, respectively. Furthermore, host cells must be found for isolating and obtaining the source DNA/RNA or protein from the viruses. Nanopore-based assays can detect unknown viruses in the absence of such biochemical information, however, based simply on virus electrical properties. Further developments in in-plane sensor technology could lead to mobile and wearable devices for monitoring infectious viruses ubiquitously, which could enhance public safety and health. From a long-term prospective, we can believe that nanopore-based virus-sensing techniques will assume a more central role in the quantification of viruses.

## Author contributions

LY wrote the draft and TY revised the manuscript.

### Conflict of interest statement

The authors declare that the research was conducted in the absence of any commercial or financial relationships that could be construed as a potential conflict of interest.
